# Transcriptome Analysis of the Ammonia-Oxidizing Bacterium *Nitrosomonas mobilis* Ms1 Reveals Division of Labor between Aggregates and Free-living Cells

**DOI:** 10.1264/jsme2.ME19148

**Published:** 2020-02-28

**Authors:** Rino Isshiki, Hirotsugu Fujitani, Satoshi Tsuneda

**Affiliations:** 1 Department of Life Science and Medical Bioscience, Waseda University, 2–2 Wakamatsu-cho, Shinjuku, Tokyo, 162–8480, Japan; 2 Biomedical Research Institute, National Institute of Advanced Industrial Science and Technology, Tsukuba Central 6, Tsukuba, Ibaraki 305–8566, Japan; 3 Research Organization for Nano & Life Innovation, Waseda University, 513, Wasedatsurumaki-cho, Shinjuku, Tokyo, 162–0041, Japan

**Keywords:** nitrification, aggregate, division of labor, RNA sequence, transcriptome

## Abstract

Bacteria change their metabolic states to increase survival by forming aggregates. Ammonia-oxidizing bacteria also form aggregates in response to environmental stresses. *Nitrosomonas mobilis*, an ammonia-oxidizing bacterium with high stress tolerance, often forms aggregates mainly in wastewater treatment systems. Despite the high frequency of aggregate formation by *N. mobilis*, its relationship with survival currently remains unclear. In the present study, aggregates were formed in the late stage of culture with the accumulation of nitrite as a growth inhibitor. To clarify the significance of aggregate formation in *N. mobilis* Ms1, a transcriptome analysis was performed. Comparisons of the early and late stages of culture revealed that the expression of stress response genes (chaperones and proteases) increased in the early stage. Aggregate formation may lead to stress avoidance because stress response genes were not up-regulated in the late stage of culture during which aggregates formed. Furthermore, comparisons of free-living cells with aggregates in the early stage of culture showed differences in gene expression related to biosynthesis (ATP synthase and ribosomal proteins) and motility and adhesion (flagella, pilus, and chemotaxis). Biosynthesis genes for growth were up-regulated in free-living cells, while motility and adhesion genes for adaptation were up-regulated in aggregates. These results indicate that *N. mobilis* Ms1 cells adapt to an unfavorable environment and grow through the division of labor between aggregates and free-living cells.

Bacteria form appropriate microstructures that increase survival in response to various environmental changes. The aggregates of multiple bacteria are considered to be the main form in natural environments and engineered systems (Davey and O’Toole, 2000; Hall-Stoodley *et al.*, 2004). These aggregates enable environmental responses as a group and facilitate interactions among cells. As an environmental response, biofilm formation may limit the diffusion of antibacterial agents, metals, and toxins. Aggregation has been shown to protect cells from various environmental stresses, such as ultraviolet (UV) irradiation, a pH shift, osmotic shock, and drying (Decho, 1990; Flemming, 1993; Gilbert *et al.*, 1997). In addition, cell-cell interactions increase the availability of nutrients by providing opportunities for metabolite exchange and the elimination of toxic metabolites (Costerton *et al.*, 1995). Furthermore, aggregates with closely attached cells increase the possibility of horizontal gene transfer through conjugation, thereby contributing to the evolution and genetic diversity of microbial communities (Davey and O’Toole, 2000).

Ammonia-oxidizing bacteria (AOB) are often observed in aggregates rather than as free-living cells (Koops *et al.*, 1991; Allison and Prosser, 1993; Verhagen *et al.*, 1994; Wagner *et al.*, 1995; Vejmelkova *et al.*, 2012). AOB are chemoautotrophic bacteria that are responsible for the conversion of ammonia to nitrite, which is an important process in the nitrogen cycle in terrestrial, aquatic, and drainage systems. Even in AOB, aggregated structures, such as biofilms, increase resistance to environmental stresses, including low pH and the presence of nitrification inhibitors (Underhill and Prosser, 1987; Powell and Prosser, 1991; Allison and Prosser, 1993; Lauchnor *et al.*, 2011). These effects are often attributed to the exopolysaccharide (EPS) produced by AOB (Cox *et al.*, 1980). When cells aggregate and become localized very close to each other at a high density, the induction period is shortened and recovery from starvation is accelerated (Armstrong and Prosser, 1988; Powell and Prosser, 1991; Batchelor *et al.*, 1997). Therefore, AOB aggregates have different physiological characteristics from free-living cells, which provide survival advantages.

*Nitrosomonas mobilis* is an AOB that often forms aggregates in activated sludge. Microscopic observations using fluorescence *in situ* hybridization previously revealed that all aggregates were stained with the NmV probe specific for the *N. mobilis* lineage (Juretschko *et al.*, 1998; Gieseke *et al.*, 2003). *N. mobilis* was identified as the dominant AOB species during the formation of nitrifying granules, which are the self-granulating aggregates of microorganisms used in wastewater treatment plants (Matsumoto *et al.*, 2010). *N. mobilis* Ms1 was isolated from nitrifying granules in the form of aggregates and formed aggregates even in pure cultures (Fujitani *et al.*, 2015). One of the distinct physiological properties of *N. mobilis* Ms1 is tolerance to high concentrations of ammonia and nitrite (Thandar *et al.*, 2016). Genomic information was also obtained (Thandar *et al.*, 2016).

Since *N. mobilis* Ms1 forms aggregates and is highly resistant to stress, it may acquire stress tolerance by forming aggregates. However, the metabolic state in which *N. mobilis* Ms1-aggregated cells create advantages for survival currently remains unclear. In the present study, a transcriptome analysis was performed on *N. mobilis* Ms1 to compare aggregates with free-living cells. The use of aggregates as a survival strategy by *N. mobilis* Ms1 was implied based on the discovery of differentially expressed genes (DEGs) between aggregates and free-living cells.

## Materials and Methods

### Strains and culture conditions

*N. mobilis* Ms1 isolated from nitrifying granules was cultured in a batch mode with mineral medium containing 2.1‍ ‍mM NH_4_Cl as previously reported (Fujitani *et al.*, 2015; Thandar *et al.*, 2016). Inorganic medium included NaCl (0.116 g L^–1^), MgSO_4_·7H_2_O (0.4‍ ‍g‍ ‍L^–1^), CaCl_2_·2H_2_O (0.073 g L^–1^), KCl (0.038 g L^–1^), KH_2_PO_4_ (0.034 g L^–1^), FeCl_2_ (0.002 g L^–1^), EDTA (0.0043 g L^–1^), MnCl_2_·4H_2_O (0.1 mg L^–1^), CoCl_2_·6H_2_O (0.024‍ ‍mg‍ ‍L^–1^), NiCl_2_·6H_2_O (0.024‍ ‍mg‍ ‍L^–1^), CuCl_2_·2H_2_O (0.017 mg L^–1^), ZnCl_2_ (0.068 mg L^–1^), Na_2_WO_4_·2H_2_O (0.033 mg L^–1^), Na_2_MoO_4_ (0.024‍ ‍mg‍ ‍L^–1^), and H_3_BO_3_ (0.062 mg L^–1^). Cells were grown at 28°C without shaking in triplicate 500-mL cultures under dark conditions. All analyzed cells were prepared by inoculating cells from the early exponential phase culture into fresh medium and then cultivation for 2‍ ‍weeks (early stage) or 8‍ ‍weeks (late stage). During the culture, ammonium concentrations were measured once every 2 to 4‍ ‍d using the indophenol method (Kandeler and Gerber, 1988). NH_4_Cl was added periodically to a final concentration of 2.1‍ ‍mM in order to avoid depletion. The pH of the medium was adjusted to 8.5 with NaHCO_3_. The concentration of the nitrite produced was measured by the Griess method (Hewitt and Nicholas, 1964). All analyzed cells were collected 24 h after the addition of NH_4_Cl and pH adjustments for microscopic observations and RNA sequencing.

### Microscopy

Cell morphology was observed using fluorescence microscopy. One milliliter of the culture was collected on weeks 2 and 8 of cultivation. The culture was sonicated with 30% amplitude for 30‍ ‍s (Q55; QSonica) and then dried on a slide glass. Cells were stained with the SYTOX Green nucleic acid stain (Thermo Fisher Scientific) and observed using a fluorescence microscope (Zeiss Axioskop 2plus, lens Zeiss Plan-APOCHROMAT 100×/1.4 oil; Carl Zeiss).

### Separation of aggregates and free-living cells

Free-living and aggregated cells were separated by filtering to compare gene expression. Approximately 500 mL of the culture was centrifuged at 4,800×*g* for 30‍ ‍min and resuspended in 10 mL of mineral medium. After sonication at 30% amplitude for 3‍ ‍min, aggregates were collected by trapping on a filter with a pore size of 5 μm (Merck). Free-living cells were collected by passage through a filter with a pore size of 5 μm and trapping on a filter with a pore size of 0.2 μm (Merck).

### Calculation of free-living and aggregated cell numbers

To calculate the cell number of *N. mobilis* Ms1, qPCR was performed targeting the *glnA* gene of DNA extracted from each sample. The *glnA* gene exists as one copy in the *N. mobilis* Ms1 genome. Therefore, the cell number matched the copy number of the *glnA* gene in extracted DNA. The primers NSMM_glnA_f (5′-GGCCATCAAGGGTGGCTATT-3′) and NSMM_glnA_r (5′-TCCACAGGGATGCCAAGTTC-3′) were used. To prepare a standard sample of the *glnA* gene, PCR was performed using TB Green Premix Ex Taq II (Tli RNaseH Plus; Takara Bio). Based on the concentration of the PCR product and length of the *glnA* gene, the copy number of the amplified *glnA* gene was calculated. The PCR product was diluted to create a standard sample. qPCR was performed by TB Green Premix Ex Taq II (Tli RNaseH Plus) and Thermal Cycler Dice Real Time System II (Takara Bio). qPCR used the following thermal profile: an initial denaturation step was conducted at 95°C for 30‍ ‍s, followed by 40 cycles of denaturation at 95°C for 5‍ ‍s, annealing at 60°C for 30‍ ‍s, and elongation at 68°C for 30‍ ‍s, with a melting curve analysis. The R^2^ value for qPCR was >0.99 (Fig. S1).

### RNA preparation and sequencing

Biological triplicates of the culture were prepared for the transcriptome analysis. To lyse cells in the culture, 100‍ ‍μL of 15‍ ‍mg‍ ‍mL^–1^ lysozyme and 10‍ ‍μL of 5 mg mL^–1^ proteinase K were added to the cell pellet collected by the filter. Since the aggregates of *N. mobilis* Ms1 strongly adhered to and were difficult to separate from and lyse cells, cells were sonicated frequently at 30% amplitude for 3‍ ‍min when aggregates were detected in subsequent operations. RNA was purified from lysates using the RNeasy Mini Kit (Qiagen). Ribosomal RNA was removed from extracted total RNA using the Ribo-Zero rRNA Removal Kit (Gram-negative bacteria; Illumina). The library was then prepared using the SureSelect Stranded Prep Kit (Agilent Technologies). The mRNA library was sequenced with Illumina HiSeq3000 under the condition of paired End 100 bp. Raw sequence data are available in the DDBJ Sequenced Read Archive under the accession number DRA007360.

### Statistical analysis

The quality of the reads was improved using CLC genomics workbench (CLC Bio) by trimming the rest of the adapter, low quality reads (limit 0.05), short reads (>15 bp), and one base from the 5′ and 3′ ends. Reads were also mapped under the conditions of‍ ‍Mismatch cost 2, Insertion cost 3, Deletion cost 3, Length fraction 0.5, and Similarity fraction 0.8 to the *N. mobilis* Ms1 genome sequence (Accession numbers FMWO01000001–FMWO01000112) (Thandar *et al.*, 2016). Based on read count data, a hierarchical clustering analysis was performed using the Bioconductor package TCC. Regarding clustering, the complete method was used as the agglomerative method, and Spearman’s rank correlation coefficient was used as the distance correlation coefficient. DEGs were normalized by the iDEGES/edgeR method using the Bioconductor package TCC, normalized by the TMM method, and tested by the edgeR method (Sun *et al.*, 2013). Genes with |logFC|>1, *P*<0.05, and a false discovery rate (FDR) <0.05 were defined as DEGs. Furthermore, DEGs were mapped to the pathway provided by the Kyoto Encyclopedia of Genes and Genomes (KEGG).

## Results and Discussion

### Ratio of aggregates and free-living cells in the long-term culture

**Fig. 1.**
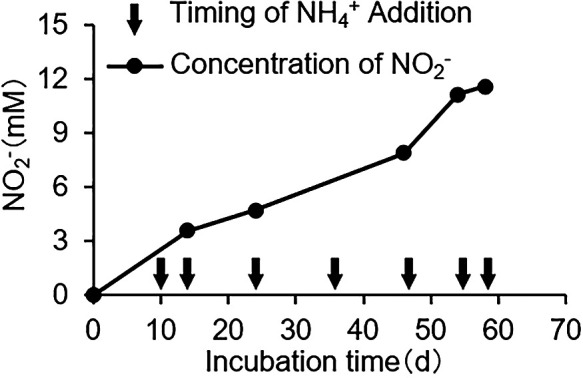
Timing of NH_4_Cl addition and nitrite accumulation during batch cultures. *N. mobilis* Ms1 was cultured for 8‍ ‍weeks with the addition of NH_4_Cl and pH adjustments. The timing of the addition of NH_4_Cl is denoted with an arrow, and the accumulation of nitrite is indicated by the line.

**Fig. 2.**
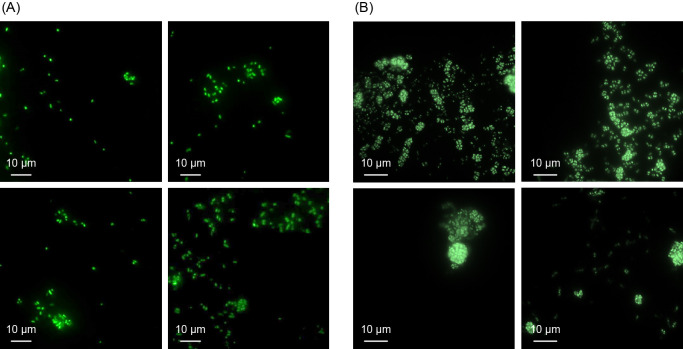
Morphological change in *N. mobilis* Ms1 in the long-term culture. *N. mobilis* Ms1 was cultured for 8‍ ‍weeks with the addition of NH_4_Cl and pH adjustments. (A) *N. mobilis* Ms1 cells in the early stage of culture (2‍ ‍weeks) and (B) late stage of culture (8‍ ‍weeks) were stained with SYTOX Green and observed by fluorescence microscopy. The scale bar indicates 10 μm.

To observe the formation of aggregates of *N. mobilis* Ms1 during cultivation, *N. mobilis* Ms1 was cultured for 8‍ ‍weeks with the addition of 2‍ ‍mM NH_4_Cl. The timing of the addition of NH_4_Cl was shown in Fig. 1. During the 8-week batch culture, NH_4_Cl was added seven times and nitrite gradually accumulated (Fig. 1). Morphological changes were observed using fluorescence microscopy. The proportion of free-living cells was high in the early stage of culture (second week; Fig. 2A). When the aggregate was defined as a structure consisting of 10 cells or more, the proportion of aggregates increased in the late stage of culture (eighth week; Fig. 2B). The difference in size of the aggregates was not large, approximately 5–10 μm between the early and late stages. The cell density of aggregates was higher in the late stage (10–50 cells per aggregate) than in the early stage (approximately 10–20 cells per aggregate). Cell numbers were quantified by qPCR (Fig. S2). In the early stage of culture, the cell numbers of free-living (FL) and aggregates (Agg) were 4.0×10^5^ and 3.7×10^5^‍ ‍cells‍ ‍mL^–1^, respectively. In the later stages of culture, the cell numbers of FL and Agg were 1.4×10^6^ and 7.0×10^8^‍ ‍cells‍ ‍mL^–1^, respectively.

During the long-time cultivation of *N. mobilis* Ms1, the proportion of aggregates was higher in the late stage than in the early stage of culture. Some AOB form self-aggregates in pure cultures in a similar manner to *N. mobilis* Ms1. For example, *N. oligotropha* forms aggregates after the exponential growth phase (Koops *et al.*, 1991; Koops and Pommerening-Röser, 2001). In addition, some AOB form aggregates when culture conditions are not favorable (Watson, 1971). A potentially unfavorable condition is a nitrite-accumulated condition. AOB produce nitrite by ammonia oxidation. However, the accumulation of nitrite inhibits cell growth (Thandar *et al.*, 2016). Instead of cell growth, these types of microbes form aggregates to survive environmental stress. Therefore, the results showing that many aggregates were observed in the late culture may be explained by the accumulation of nitrite during the long-term culture.

### Overview of transcriptome profiles

A transcriptome analysis of *N. mobilis* Ms1 was performed on early culture free-living cells (early FL), early culture aggregates (early Agg), late culture free-living cells (late FL), and late culture aggregates (late Agg). Among 3,254 *N. mobilis* Ms1 genes, the reads obtained were mapped at 3,152 genes. The overall metabolic status of the four groups was compared by clustering using read count data from the four groups of biological triplicates. As a result, late FL and late Agg were found to have similar metabolic states. There were only three DEGs (NSMM150008, NSMM260010, and NSMM260055) between late FL and late Agg. All three genes were up-regulated in late Agg; however, their functions currently remain unknown. Based on the results of the clustering analysis, four groups (early FL, early Agg, late FL, and late Agg) were roughly divided into three groups. Cells were expected to have similar metabolic states in the late stage of culture even if cell morphologies differed. In contrast, since early FL and early Agg exhibited completely different transcriptional states, morphological differences in the early stages of culture were expected to reflect different metabolic states and survival strategies.

Based on the overall metabolic status of the four groups, the two groups described below were compared. Comparisons of early FL and early Agg was performed to reveal gene expression that represented differences in morphology. Comparisons of early Agg and late Agg were performed to reveal gene expression, which represented differences in the culture period.

Comparisons of early FL and early Agg revealed the significantly increased expression of 72 genes in early FL and of 251 genes in early Agg. Comparisons of early Agg and late Agg revealed the significantly up-regulated expression of 174 genes in early Agg and of 43 genes in late Agg. The number of strongly expressed genes was higher in early Agg.

**Fig. 3.**
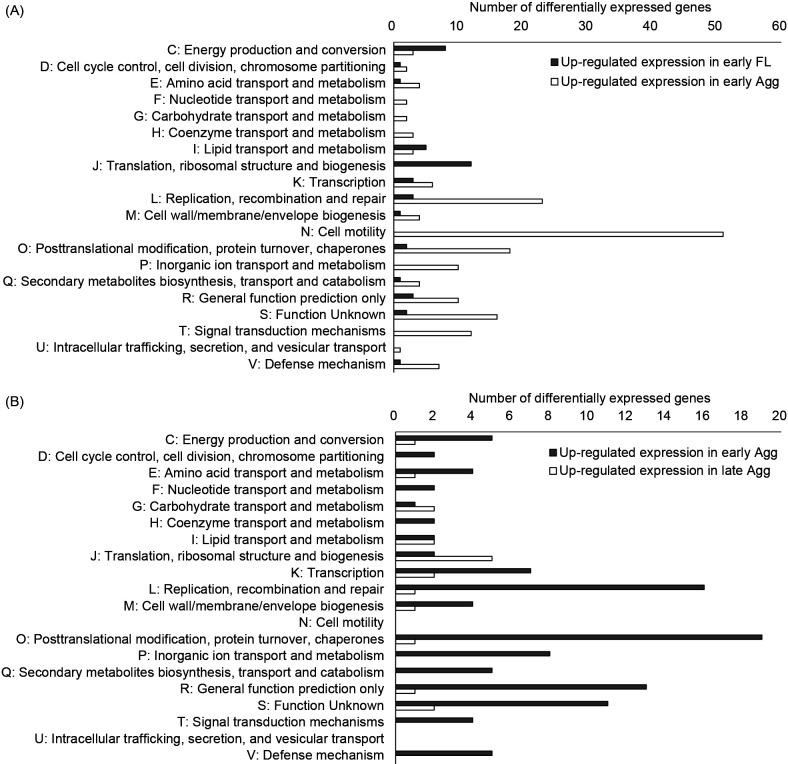
Clusters of Orthologous Groups (COG) classification of expression variable genes. COG assignment of differentially expressed genes (DEGs). Gene expression between the two groups was compared. DEGs were indicated by |‍log_2_FC|>1, *P*<0.05, and a false discovery rate (FDR) <0.05. (A) DEGs in the comparison of early FL and early Agg. Black bars indicate genes up-regulated in early FL. Gray bars indicate genes up-regulated by early Agg. (B) DEGs in the comparison of early Agg and late Agg. The black bar denotes genes whose expression was increased by early Agg. The gray bar denotes genes whose expression was increased by late Agg.

Strongly expressed genes were classified into functional clusters (Clusters of Orthologous Groups, COG) (Fig. 3). In comparisons of early FL and early Agg, many of the genes that were up-regulated in early FL were classified into energy production and conversion (category C) and translation, ribosomal structure, and biogenesis (category J). Many of the up-regulated genes in early Agg were classified into cell motility (category N) (Fig. 3A). In comparisons of early Agg and late Agg, many of the genes up-regulated in early Agg were classified into replication, recombination, and repair (category L) and posttranslational modification, protein turnover, and chaperones (category O). Many of the genes up-regulated in late Agg were classified in translation, ribosomal structure, and biogenesis (category J) (Fig. 3B).

Gene expression differences between early Agg and early FL and between late Agg and early Agg are summarized in Table 1. Table S1 lists the expression of all genes in either group. In Table S1, significantly up-regulated genes were indicated in red and down-regulated genes in blue.

### Genes related to energy production and biosynthesis

Comparisons of early FL and early Agg revealed that genes up-regulated in early FL included ATP synthases (*atpH*, *atpG*, and *atpC*), which were classified into energy production and conversion. In addition, several ribosomal proteins (*rplI*, *rpsR*, and *rpsF*) were classified into translation, ribosomal structure, and biogenesis. Biosynthesis-related gene expression was higher in early FL when morphology was different. In contrast, comparisons of early Agg and late Agg showed that genes up-regulated in late Agg included ribosomal proteins (*rpsR*, *rpsF*, and *rplP*), which were classified into translation, ribosomal structure and biogenesis. The operon encoding ribulose-1,5-bis-phosphate-carboxylase/oxygenase (RuBisCO), which is the carbon fixation pathway of *N. mobilis* Ms1, was up-regulated in late Agg. When the early and late stages were compared, the expression of the biosynthetic system increased in the late stage.

*N. mobilis* Ms1 is a chemoautotrophic microorganism that acquires energy by oxidizing ammonia using oxygen as an electron acceptor and acquires a carbon source by fixing carbon dioxide using the Calvin cycle. Most of the genes related to ammonia monooxygenase and hydroxylamine oxidoreductase (*amoA1*, *amoA2*, *amoB1*, *amoB2*, *amoC1*, *amoC2*, *amoD*, *haoA1*, *haoA2*, *haoB1*, *haoB2*, *nirK*, and NSMM680003), which are essential genes for ammonia oxidation, were strongly expressed in all groups. However, no significant differences were observed in expression. Previous AOB studies reported that *amoA* and *amoC* mRNAs were conserved under substrate starvation conditions (Wei *et al.*, 2004; Bollmann *et al.*, 2005; Wei *et al.*, 2006; Berube and Stahl, 2012; Sedlacek, *et al.*, 2020. Transcriptomic response of *Nitrosomonas europaea* transitioned from ammonia- to oxygen-limited steady-state growth. *bioRxiv* 765727). These transcriptional states suggest that the expression of genes encoding AMO and HAO, the most important enzymes for energy conversion, are always maintained. AOB recovers quickly from stress by maintaining the expression of the most important enzymes, even during stress, and *N. mobilis* Ms1 showed similar expression profiles.

In addition, genes encoding *amoE1* and *amoE2* were expressed in a different manner than the other ammonia oxidation-related genes. The *amoE* gene is harbored by beta-proteobacterial AOB and is considered to be derived from *amoD* duplication. The function of *amoE* currently remains unknown. However, structural predictions suggest that it interacts with ammonia oxidation and electron transport (El Sheikh *et al.*, 2008). Furthermore, previous studies demonstrated that *amoE* was up-regulated during recovery from ammonia starvation (Berube and Stahl, 2012). Thus, since *amoE1* and *amoE2* were up-regulated in early Agg, during which the stress response mechanism functioned, the *amoE1* and *amoE2* genes were expected to allow for the fast recovery of ammonia-oxidizing activity in *N. mobilis* Ms1.

The oxidation of ammonia to nitrite was previously considered to occur by the activity of the AMO and HAO enzymes. However, in recent years, a third enzyme has been suggested to be involved in ammonia oxidation (Caranto and Lancaster, 2017). A new gene considered to play a role in ammonia oxidation is the gene encoding nitrosocyanin. This gene is conserved in most AOB and is expressed at the same level as AMO and HAO (Whittaker *et al.*, 2000; Zorz *et al.*, 2018). In this study, the gene nitrosocyanin was expressed similar to *amoCAB* and *haoAB*. Another candidate third enzyme is *nirK*. The *nirK* gene mutant of *N. europaea* was shown to have a lower growth rate, lower cell yield, and higher sensitivity to NO_2_^–^ in an aerobic culture than the wild type (Schmidt *et al.*, 2004; Beaumont *et al.*, 2005). NirK also functions as an electron sink that promotes efficient NH_2_OH to NO_2_^–^ oxidation (Cantera and Stein, 2007). In this study, the *nirK* gene was also expressed similarly to *amoCAB* and *haoAB*. Thus, these genes are more likely to be candidates as the third enzyme.

### Genes related to cell motility

Comparisons of early Agg to early FL revealed the significant up-regulation of genes related to cell motility in early Agg. The up-regulated genes in early Agg included flagellar genes (*fliG*, *fliC*, and *fliL*), pilus genes (*pilT*, *pilM*, and *pilN*), chemotaxis genes (*cheY*, *cheA*, and *cheW*), and two-component genes (*fleS* and *yrbD*). The EPS biosynthetic gene NSMM150022, which is involved in cell-cell adhesion (Davey and O’Toole, 2000), was up-regulated in early Agg. A gene pathway analysis and mapping using the KEGG mapper predicted that the majority of the flagellar genes were up-regulated in early Agg and that entire flagella were composed.

Comparisons of early FL and early Agg demonstrated that the expression of genes related to motility, chemotaxis, and EPS was markedly stronger in early Agg. Regarding the flagellar activity of AOB, a comparative proteomic analysis of *N. europaea*, *N. ureae*, and *Nitrosospira multiformis* showed that the expression of flagellar proteins only increased in *N. multiformis* (Zorz *et al.*, 2018). Since flagellar synthesis is observed in species with high aggregation abilities, such as *N. multiformis* and *N. mobilis* Ms1, a relationship may exist between aggregation ability and flagellar synthesis activity.

This result indicates that motility, chemotaxis, and EPS synthesis are related to aggregate formation. Similar to other AOB aggregates (Allison and Prosser, 1993; Stehr *et al.*, 1995; Matsumoto *et al.*, 2010), *N. mobilis* Ms1 may also synthesize EPS. Although the motility of aggregated cells may appear to be unnatural, motility is often observed in the early stages of biofilm formation. Small aggregates that resemble microcolonies are formed in the early stages of biofilm development (Stoodley *et al.*, 2002). Microcolony cells have motility to repeat adhesion and spreading. Flagella, type IV pili, and EPS synthesis are important for this motility during the early stages of biofilm formation (O’Toole and Kolter, 1998; Davey and O’Toole, 2000). The genes related to flagella, type IV pili, and EPS synthesis were also up-regulated in *N. mobilis* Ms1, which suggests that aggregates of *N. mobilis* Ms1 behave in a similar manner to cells in the early stages of biofilm formation.

Furthermore, the expression status of whole transcripts showed that cells in the late stage culture possessed aggregate properties even if cells were present in a free-living state. This may be explained by the hypothesis that free-living cells in the late culture may repeat adhesion and spreading with aggregates. It is important to note that the factors causing adhesion and spreading may be physical stimuli through experimental manipulations in addition to the properties of cells for expanding aggregates such as biofilms.

Regarding the mechanisms controlling motility, previous studies demonstrated that the prophage region CP4-57 increased the expression of motility operons, such as *flg*, *flh*, and *fli*, in *Escherichia coli* (Wang *et al.*, 2009). In *N. mobilis* Ms1 aggregates, the expression of the CP4-44 prophage region was up-regulated and, thus, may be involved in the regulation of the motor operon.

### Genes related to stress tolerance and gene transfer

Comparisons of early Agg and late Agg revealed the up-regulation of genes encoding refolding proteins, including chaperones (*dnaJ*, *clpB*, and *dnaK*) and proteases (*lon*, MSNN340021, and *clpA*) in early Agg. In contrast to the accumulation of the growth inhibitor nitrite in the long-term culture, the increased expression of stress response genes was not confirmed in the late stage of culture. Instead, the expression of stress response genes was increased in the early stage of culture. This result may be explained by assuming that *N. mobilis* Ms1 is always exposed to stress, and that aggregate formation leads to stress protection. In the early stage of culture, cells may be exposed to stress, leading to the need for adaptation. In contrast, in the late stage of culture, since many cells form aggregates and may avoid stress, there may be no need for a stress response.

Comparisons of early Agg to other groups revealed the up-regulation of genes related to defense mechanisms against foreign DNA and RNA, such as CRISPR-related genes (NSMM260103, NSMM260105, and *cas*) and restriction-modification systems (NSMM160022, NSMM480070, and *hsdR*) in early Agg. In addition, the expression of transposons and conjugation genes (NSMM70001, NSMM330006, and *int*) was up-regulated in early Agg. Based on these results, early Agg may uptake many foreign genes. Horizontal gene transfer mechanisms are generally related to the mechanisms of stress resistance, such as the acquisition of antibiotic resistance and bioremediation ability (Mahillon and Chandler, 1998). In early cultures in which the stress response is active, horizontal gene transfer may be used to increase stress tolerance by acquiring foreign genes. Furthermore, horizontal gene transfer may also be involved in the acquisition of stress tolerance in assemblies that adapt to the environment through motility and chemotaxis.

### Division of labor between aggregates and free-living cells

**Fig. 4.**
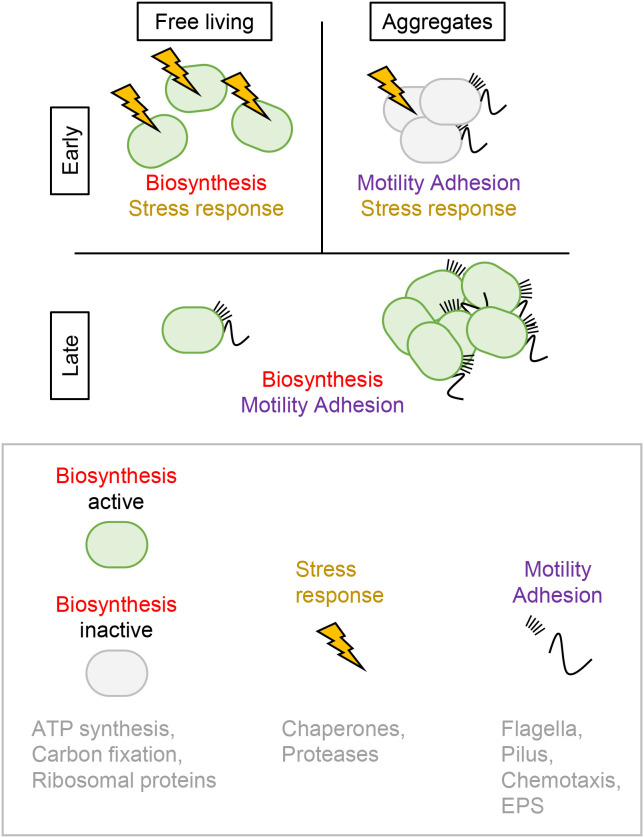
Summary of main differentially expressed genes. Differentially expressed genes were categorized into three groups: stress response systems (chaperones and proteases), biosynthesis systems (ATP synthesis, carbon fixation, and ribosomal proteins), and motility and adhesion systems (flagella, pilus, chemotaxis, and EPS). Stress response systems were strongly up-regulated in the early stages of culture, regardless of morphology. Biosynthesis systems were strongly up-regulated in early free-living cells and the late stage of culture. Motility and adhesion systems were strongly up-regulated in aggregates regardless of the culture period.

The main gene expression states are summarized in Fig. 4. Genes were categorized into three groups: stress response systems (chaperones and proteases), biosynthesis systems (ATP synthesis, carbon fixation, and ribosomal proteins), and motility and adhesion systems (pilus, flagella, chemotaxis, and EPS). Stress response systems were strongly up-regulated in the early stages of culture, regardless of morphology. Biosynthesis systems were strongly up-regulated in early free-living cells and the late stage of culture. Genes involved in the motility and adhesion system were strongly up-regulated in aggregates regardless of the culture period. These results indicate that a stress response is required in the early stages of culture. Furthermore, free-living cells may be responsible for growth-related biosynthetic systems, while aggregates are responsible for environmental adaptation with motility and adhesion. The clear differences observed in gene expression patterns between free-living cells and aggregates suggest that labor is divided between cells with two types of subpopulations, free-living cells and aggregates, in the early stage of culture. In other words, *N. mobilis* Ms1 cell populations are divided into the growth mode and environmental adaptation mode. These two distinct modes realize both population expansion through growth and species survival through adaptation to changing environments.

Regarding the division of labor, *Salmonella enterica* subsp. Enterica serovar Typhimurium has been shown to divide the cell population into two subpopulations during its infection of animal cells (Ackermann *et al.*, 2008; Diard *et al.*, 2013). One subpopulation remains in the intestinal lumen and grows, while the other subpopulation enters the intestinal tissue and enhances bactericidal effects by stimulating the host immune system. The stimulation of the host immune system appears to be a negative effect for *Salmonella* themselves. However, it may eliminate other bacteria. Thus, the *Salmonella* population may be divided into growth and killer subpopulations. As a result, only *Salmonella* survives in animal cells. Similar examples have been reported for many microorganisms, such as *Dictyostelium discoideum* and *Myxococcus xanthus* (Strassmann *et al.*, 2000; Velicer *et al.*, 2000). Thus, the division of growth and adaptation modes within one species is the primary survival strategy for microorganisms (West and Cooper, 2016). In contrast, in the late stage of the *N. mobilis* Ms1 culture, stress responses were no longer necessary. Thus, we predict that aggregates grow and adapt in parallel. *N. mobilis* Ms1 may change its life modes depending on environmental conditions in order to maintain the balance between growth and adaptation.

## Conclusion

The transcriptome analysis of *N. mobilis* Ms1 suggested that cells change their morphology by altering gene expression in response to the environment. When stress responses are required, as in the early stage of culture, genes related to biosynthesis are up-regulated in free-living cells and genes related to adaptation are up-regulated in aggregates. When a stress response is not required, as in the case of the late stage of culture, genes related to biosynthesis and adaptation are up-regulated at the same time, regardless of morphology. The present results indicate that two subpopulations of *N. mobilis* Ms1 with different phenotypes divide labor, such as growth and adaptation, particularly when stress responses are required. The formation of aggregates, which are considered to be the main forms of environmental microorganisms, reflect the survival strategy of *N. mobilis* Ms1.

## Citation

Isshiki, R., Fujitani, H., and Tsuneda, S. (2020) Transcriptome Analysis of the Ammonia-Oxidizing Bacterium *Nitrosomonas mobilis* Ms1 Reveals Division of Labor between Aggregates and Free-living Cells. *Microbes Environ ***35**: ME19148.

https://doi.org/10.1264/jsme2.ME19148

## Supplementary Material

Supplementary Material 1

Supplementary Material 2

## Figures and Tables

**Table 1.** Gene expression changes between early Agg and early FL and between late Agg and early Agg

Category and Genes				Log_2_FC	
Label	Product	COG		earlyAgg /earlyFL	lateAgg /earlyAgg
**Ammonia oxidation-related genes**					
0350049	amoE1, conserved exported protein of unknown function			0.99	–1.26
0880004	amoE2, conserved exported protein of unknown function			1.01	–1.25
**Energy production and conversion**					
0140010	atpH, ATP synthase subunit delta	C		–1.15	0.81
0140012	atpG, F1 sector of membrane-bound ATP synthase, gamma subunit	C		–1.02	0.73
0140014	atpC, F1 sector of membrane-bound ATP synthase, epsilon subunit	C		–1.03	0.49
**Translation, ribosomal structure, and biogenesis**					
0110006	rplI, 50S ribosomal subunit protein L9	J		–1.31	0.89
0110007	rpsR, 30S ribosomal protein S18	J		–1.68	1.31
0110009	rpsF, 30S ribosomal protein S6	J		–1.43	1.17
0360010	rplP, 50S ribosomal protein L16	J		–1.09	1.10
**C fixation**					
0380014	cbbS, Ribulose bisphosphate carboxylase small chain			–0.51	1.36
0500025	cbbX, Protein CbbX	O		0.57	1.80
0500026	cbxSP, Ribulose bisphosphate carboxylase small chain, plasmid			0.41	2.67
0500027	cbbL, Ribulose bisphosphate carboxylase large chain	G		0.47	2.55
**Cell motility**					
0150046	fliG, flagellar motor switching and energizing component	N		2.08	–0.13
0150048	fleS, putative two-component sensor	T		2.30	–0.18
0150060	fliC, Flagellin	N		2.41	–0.15
0150140	fliL, putative flagellar fliL transmembrane protein	N		2.29	0.05
0310032	pilT, Twitching motility protein	N		1.89	0.08
0380017	cheY, chemotaxis regulator transmitting signals to the flagellar motor component	T		1.30	0.08
0380032	pilM, putative type 4 fimbrial biogenesis protein PilM	N		2.42	–0.27
0380033	pilN, putative type 4 fimbrial biogenesis protein PilN	N		2.22	–0.33
0480032	cheA, fused chemotactic sensory histidine kinase in the two-component regulatory system	N		2.52	–0.18
0480033	cheW, purine-binding chemotaxis protein	N		1.88	0.53
0800052	yrbD, toluene transporter subunit: membrane component of the ABC superfamily	Q		1.12	–1.04
**Exopolysaccharide**					
0150022	Exopolysaccharide synthesis, ExoD			1.44	–0.93
**Posttranslational modification, protein turnover, chaperones**					
0150067	dnaJ, chaperone Hsp40, co-chaperone with DnaK	O		1.89	0.04
0150019	clpB, protein disaggregation chaperone	O		2.20	–3.58
0150066	dnaK, chaperone Hsp70 in DNA biosynthesis/cell division	O		1.72	–2.61
0250024	lon, DNA-binding ATP-dependent protease La	O		1.94	–2.92
0340021	Serine proteases, subtilase family	O		2.29	–0.86
0380114	clpA, ATPase and specificity subunit of ClpA-ClpP ATP-dependent serine protease, chaperone activity	O		1.13	–1.16
**CRISPR and restriction-modification system**					
160022	Type I restriction-modification system, M subunit	V		1.59	–2.00
260103	CRISPR-associated helicase Cas3	R		2.37	–0.50
260105	CRISPR-associated protein, Csd1 family			1.67	–0.35
0260111	cas, CRISPR-associated endoribonuclease Cas2 3	L		1.12	–0.92
480070	Type III restriction enzyme, res subunit	V		1.08	–1.03
0480084	hsdR, Type-1 restriction enzyme R protein	V		1.01	–0.81
**Gene transfer**					
70001	transposase	L		1.32	–1.71
330006	Type-F conjugative transfer system protein TraW			1.78	–1.08
330008	Type-F conjugative transfer system pilin assembly protein TrbC			1.21	–0.86
330050	TraB pilus assembly	E		1.86	–1.30
0380006	int, Integrase/recombinase	L		1.32	–1.00
0540044	insH, IS5 transposase and trans-activator; CP4-44 prophage	L		1.13	–0.95
