# Supplementary material for: Transcriptome Analysis of the Ammonia-Oxidizing Bacterium Nitrosomonas mobilis Ms1 Reveals Division of Labor between Aggregates and Free-living Cells
Source: Microbes Environ. 2020 Feb 28;35(2):ME19148. doi: 10.1264/jsme2.ME19148 (PMC7308568; doi:10.1264/jsme2.ME19148)
Supplement: Supplementary file 1 — Supplementary Material 1 [file 35_19148_s1.pdf]

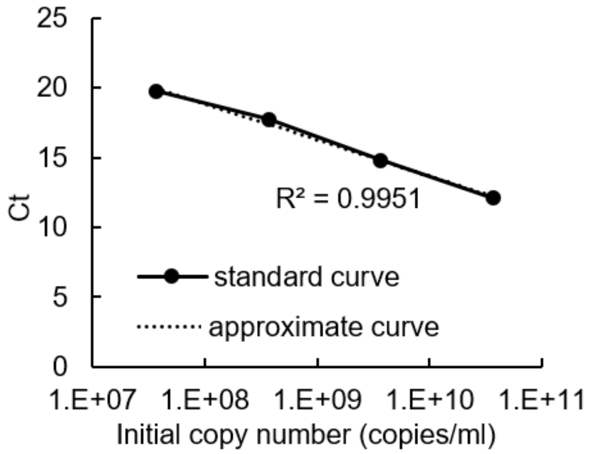

Fig. S1 The standard curve used in the qPCR

To show the accurate cell number of free-living cells and aggregates, we conducted qPCR experiments based on *glnA* gene. The *glnA* gene is existed as one copy in *N. mobilis* Ms1 genome. Therefore, the cell number matched the copy number of the *glnA* gene in the extracted DNA. The qPCR data was highly credible (R^2 value > 0.99)

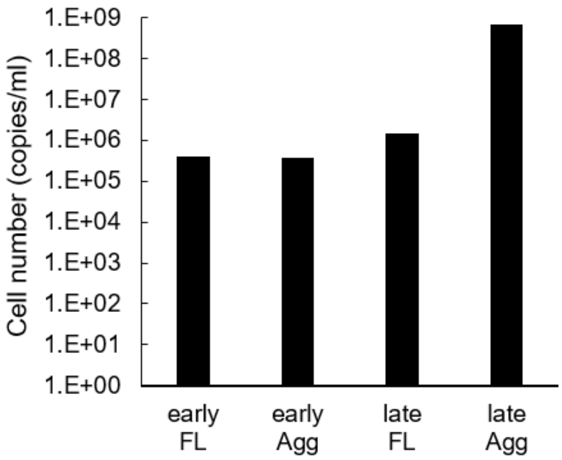

Fig. S2 The cell densities in each sample

As a result of the qPCR, early FL and early Agg were present in almost the same ratio. In contrast, late Agg were present approximately 100 times more than late FL.
